# Hybridization in a warmer world

**DOI:** 10.1002/ece3.1052

**Published:** 2014-04-16

**Authors:** Amanda J Chunco

**Affiliations:** Department of Environmental Studies, Elon UniversityCB 2015, Elon, North Carolina 27244

**Keywords:** Global change, hybrid zone dynamics, mate choice, phenology, species distribution

## Abstract

Climate change is profoundly affecting the evolutionary trajectory of individual species and ecological communities, in part through the creation of novel species assemblages. How climate change will influence competitive interactions has been an active area of research. Far less attention, however, has been given to altered reproductive interactions. Yet, reproductive interactions between formerly isolated species are inevitable as populations shift geographically and temporally as a result of climate change, potentially resulting in introgression, speciation, or even extinction. The susceptibility of hybridization rates to anthropogenic disturbance was first recognized in the 1930s. To date, work on anthropogenically mediated hybridization has focused primarily on either physical habitat disturbance or species invasion. Here, I review recent literature on hybridization to identify how ecological responses to climate change will increase the likelihood of hybridization via the dissolution of species barriers maintained by habitat, time, or behavior. Using this literature, I identify several cases where novel hybrid zones have recently formed, likely as a result of changing climate. Future research should focus on identifying areas and taxonomic groups where reproductive species interactions are most likely to be influenced by climate change. Furthermore, a better understanding of the evolutionary consequences of climate-mediated secondary contact is urgently needed. Paradoxically, hybridization is both a major conservation concern and an important source of novel genetic and phenotypic variation. Hybridization may therefore both contribute to increasing rates of extinction and stimulate the creation of novel phenotypes that will speed adaptation to novel climates. Predicting which result will occur following secondary contact will be an important contribution to conservation for many species.

## Introduction

As early as the 1930s, biologists recognized that anthropogenic disturbance could result in hybridization between two previously isolated species (Wiegand 1935; Riley 1938). In his seminal paper, Anderson (1948) outlined how anthropogenic disturbance could modify the habitat in such a way that hybridization was both more likely to occur and hybrid offspring more likely to survive. He coined the phrase “hybridization of the habitat” to describe this process (Anderson 1948). Work on this topic has advanced greatly over the last 60 years, and hybridization resulting from anthropogenic disturbance is now recognized as a major conservation concern for many species (Rhymer and Simberloff 1996; Allendorf et al. 2001). Since Anderson, research on anthropogenically mediated hybridization has focused primarily on either physical modifications to the habitat (e.g., Kimura and Munehara 2010) or on the intentional and unintentional introduction of exotic species (Rhymer and Simberloff 1996; Walters et al. 2008). However, anthropogenic global climate change is currently resulting in wide-scale habitat modification that will result in increasing opportunities for hybridization in a manner analogous to other forms of anthropogenic disturbance.

The long-term ecological consequences of climate change are profound and range from the decoupling of species interactions (Visser and Both 2005) to the restructuring of entire ecosystems (Williams and Jackson 2007; Traill et al. 2010). In addition to altering ecological processes, climate change will have consequences for evolutionary processes. Many species will face altered selection regimes in novel climates. To avoid extinction, species must acclimate, adapt, or disperse to more favorable environments. Each of these processes will influence the evolution of both individual species and communities. Although substantial research effort has examined community change resulting from climate change (e.g., Kardol et al. 2010; Yang et al. 2011), these studies have primarily focused on exploitative interactions such as the decoupling of predator–prey phenology (e.g., Van Der Jeugd et al. 2009). Relatively few studies have explicitly considered how climate change affects interspecific reproductive interactions (but see Garroway et al. 2010; Wellenreuther et al. 2010). Yet, because climate change is reshuffling species assemblages and is breaking down physical, temporal, and behavioral reproductive barriers between species, climate change is likely to dramatically influence the likelihood of hybridization in future communities.

In this review, I argue that climate change will have important evolutionary consequences for many species and that the role of climate change in shaping reproductive interactions demands more attention. Both premating and postmating barriers will be altered by climate change. Isolating barriers are critical in maintaining genetically unique populations. Increasing rates of hybridization may result in the extinction of unique populations or species through unsuccessful reproductive effort or via introgression with a more common species (Rhymer and Simberloff 1996; Allendorf et al. 2001). Hybridization may therefore increase the speed of extinction resulting from climate change. At the same time, hybridization is a major source of evolutionary innovation (Arnold 1997; Rieseberg et al. 2003) and offers the opportunity for phenotypic and genetic novelty at a pace much faster than within-species adaptation. Thus, hybridization between populations adapted to disappearing climates and those already adapted to emerging climates may allow some species to survive in rapidly changing environments. Below, I review several recent examples of the effects of climate change on the movement and formation of hybrid zones.

## PreMating Barriers

### Spatial isolation

Temperature is a major driver of both latitudinal and altitudinal range boundaries (Merriam 1894; Gaston 2003) and habitat use (Knowlton and Graham 2010). Recent global temperature increases have resulted in documented range shifts for hundreds of species (Parmesan et al. 1999; Thomas and Lennon 1999; La Sorte and Thompson 2007). Range dynamics of even closely related species are rarely affected by climate change in the same way or at the same rate (Tingley et al. 2009): individual species tend to show significant variation in response to climate change in terms of range displacement when compared within a single taxonomic group (Angert et al. 2011) or even within a single genus (Moritz et al. 2008). This differential response between closely related allopatric or allotopic species can result in secondary contact and the formation of a novel hybrid zone.

Novel hybrid zones will form if climate change removes habitat barriers between currently allopatric sister taxa. One dramatic example of this type of habitat change is the melting of Arctic sea ice that is predicted to bring many formerly isolated taxa into contact (Kelly et al. 2010; Heide-Jørgensen et al. 2012). New cases of hybridization resulting from recent climate-mediated shifts in range boundaries are already emerging (Table 1). For example, a new hybrid zone between southern flying squirrels and northern flying squirrels (*Glaucomys volans x G. sabrinus*) formed following a 200 km northern range shift by southern flying squirrels into the range of northern flying squirrels within the last 15 years (Garroway et al. 2010). Similarly, recent hybridization between brown hares and mountain hares (*Lepus europaeus x L. timidus*) is likely the result of a range expansion by brown hares northwards into formerly allopatric mountain hare habitat (Jansson et al. 2007).

**Table 1:** Empirical or experimental cases of hybrid zones potentially affected by climate change

Example	Species	Taxonomic class	Isolating barrier	Geographic region	Time period of study	Current conservation concern	References
1	*Lagopus lagopus x L. muta*	Aves	Spatial	Sweden	Samples collected 2005–2007	Potentially; one species is declining	Quintela et al. (2010)
2	*Glaucomys volans x G. sabrinus*	Mammalia	Spatial	Pennsylvania, USA and Ontario, Canada	Field surveys 2002–2004; comparison to northern range limit in 1988	Yes; *G. sabinus* is locally endangered	Garroway et al. (2010)
3	*Lepus europaeus x L. timidus*	Mammalia	Spatial, behavioral	Sweden	Field surveys from 2003 to 2005; new brown hare populations became established with onset of milder winters in 1987	Potentially; *L. timidus* is declining	Jansson et al. (2007)
4	*Two isolated populations of Brassica rapa*	Magnoliopsida	Temporal	Field collected seeds from California, USA	Seeds collected in 1997 and 2004	No	Franks and Weis (2009)
5	*Calopteryx splendens x C. virgo*	Insecta	Spatial	Sweden and Finland	Unknown	No	Wellenreuther et al. (2010)
6	*Plethodon teyahalee x Plethodon shermani*	Amphibia	Spatial	North Carolina, USA	Study evaluated movement between 1974 and 1990	Potentially; *P. shermani* is vulnerable	Walls (2009)
7	*Allonemobius fasciatus x A. socius*	Insecta	Spatial	Appalachian Mountains, USA	1986–1999	No	Britch et al. (2001)
8	*Pyrola minor x P. grandiflora*	Magnoliopsida	Spatial	Greenland, Canada, Estonia, and Sweden	Unspecified; recent field surveys with qualitative comparison to samples from 1950s	Potentially; *P. minor* is locally rare and may have limited ability to track habitats because of hybridization with *P. grandiflora*	Beatty et al. (2010)
9	*Papilio canadensis x P. glaucus*	Insecta	Temporal	Northern continental USA and Alaska	1992–1995	No	Scriber (2002)
10	*Strix varia x S. occidentalis caurina*	Aves	Spatial	Washington and Oregon, USA	1974–1999	Yes; *S. occidentalis caurina* is threatened	Kelly et al. (2003)
11	*Ipomopsis aggregata x I. tenuituba*	Magnolopsida	Postzygotic barriers	Colorado, USA	2009–2011	No	Campbell and Wendlandt (2013)
12	*Daphnia galeata x D. hyalina*	Branchiopoda	Postzygotic barriers	Saxony, Germany	2005–2007	No	Zeis et al. (2010)
13	*Ambystoma tigrinum x A. californiense*	Amphibia	Postzygotic barriers	California, USA	Unknown	Yes, *A. californiense* is endangered	Johnson et al. (2010)

In general, as species track suitable habitat, a new contact zone will form when the leading edge of an advancing southern (or lower latitude) taxa advances faster than the lagging edge of a retreating northern (or higher latitude) sister taxa resulting in contact at the range margins. Whether or not there is a general expectation that leading edges will expand faster than lagging edges is currently unclear. A few studies have suggested that northern edges are expanding more quickly than southern edges have retracted. For example, Parmesan et al. (1999) have found that a higher percentage of European butterfly species have undergone a range expansion at the northern border than those that have undergone a range retraction at the southern border. Also in butterflies, Chen et al. (2011) found that upper elevation boundaries increased faster than lower boundaries retreated. In general, we would expect the leading edge to expand faster than the trailing edge retracts if northern range limits are more sensitive to climate or if the speed of climate change is faster at northern range limits (Parmesan et al. 1999). This pattern is, however, not universal. Two recent studies independently used USDA Forest Service Forest Inventory and Analysis data to demonstrate that southern range retractions are more common than northern range expansions in eastern United State tree species (Murphy et al. 2010; Zhu et al. 2012). Whether or not the leading edge will typically expand at a faster rate than the lagging edge retracts is likely to be highly taxon specific. Species with life-history traits related to high dispersal and colonization potential will likely show a rapidly expanding leading edge, and thus the potential to form new hybrid zones, while poor colonizers will have a slower relative rate of range expansion and thus have lower potential to form new hybrid zones. More study on the relative population dynamics in leading and lagging edge populations are needed both to better understand the response to climate change (Hampe and Petit 2005) and to predict the taxa most likely to form new hybrid zones.

When leading edge expansion outpaces lagging edge retraction, new contact zones are likely to result. These contact zones may be relatively permanent: If the lagging edge remains stable, the future of the two populations will then depend on the relative fitness of hybrid offspring. Alternatively, these contact zones might be temporary if the lagging edge does eventually retract as has occurred in past periods of climate change. For example, molecular evidence suggests previous climatic cycles resulted in the formation and dissolution of hybrid zones in several species including mountain hares (*Lepus timidus*) (Melo-Ferreira et al. 2007) and baboons (*Papio spp*.) (Zinner et al. 2009). Furthermore, previous periods of glaciation and deglaciation likely promoted secondary contact between *Arabidopsis lyrata* and *A. halleri*, resulting in the formation of a third, hybrid, allopolyploid species, *A. kamchatica* (Schmickl et al. 2010).

Recent biogeographic studies provide strong evidence for the importance of climate on hybrid zone location and formation. Hybridizing populations often occur at the range edges of the two parental species (Britch et al. 2001; Bridle and Vines 2007), and the boundaries of these contact hybrid zones are driven by climate (Swenson and Howard 2005; Swenson 2006). For example, temperature is important in maintaining the geographic location of four avian hybrid zones (Swenson 2006). Several recent studies have implicated climate change in hybrid zone movement (Buggs 2007). For example, the movement of a salamander hybrid zone (*Plethodon jordani x P. glutinosus*) in the Smokey Mountains had previously been attributed to the end of intensive logging (Hairston et al. 1992), but additional analyses indicate that movement may instead be the result of increasing temperature in this region (Walls 2009). The northward shift of a cricket hybrid zone (*Allonemobius fasciatua x A. socius*) as the southern parental species expands its range may also be driven by climate change (Britch et al. 2001).

Although most recent attention has focused on shifts in species distributions at range edges, novel reproductive interactions could occur even without shifting range margins. A growing body of evidence shows that many individual species are shifting their microhabitat usage in response to climate change (e.g., Davies et al. 2006). Recent research suggests that global climate change influences species interactions at these small scales. For example, Martin (2001) found that ground nesting birds tracked microclimates even at the expense of moving away from preferred vegetation types, which resulted in increased predation pressure. As with shifts in range boundaries, shifts in microhabitat use may alter species interactions and results in hybridization.

Shifting microhabitat usage may influence the likelihood of hybridization via several general mechanisms. First, if species are pushed into suboptimal habitat in order to track shifting microclimates, species that were previously segregated by microhabitat differences may come into contact. Bickford et al. (2010) has suggested that many amphibian species will shift microhabitat use to cooler environments and that the reduction in available suitable microhabitats may force species into more crowded conditions. Increased crowding can lead to increased instances of hybridization in amphibians (Simovich 1985). Alternatively, climate change may result in species *increasing* their total microhabitat usage. For example, silver-spotted skipper butterflies (*Hesperia comma*) have increased the amount of area occupied because their total habitat breadth has increased over the last 20 years (Davies et al. 2006). An increase in the types of habitats used could bring two species that were formerly segregated by microhabitat differences into secondary contact. Finally, host plant usage by many insect species is driven, in part, by climate, with individuals preferring host species that provide a cooler microhabitat in warmer ambient conditions (Ashton et al. 2009). Host plant usage is a reproductive barrier for many insect species (Coyne and Orr 2004). In all these cases, climatic changes resulting in shifting microhabitat use may result in novel instances of hybridization.

Thus far, most research attention has focused on range edges, as range edges are often well-known and documenting range expansions is relatively straightforward. Microhabitat usage is more subtle and occurs at smaller spatial scales than most distribution maps record (Thomas and Abery 1995). Therefore, changes in microhabitat use are less likely to be observed. As pointed out by Thomas et al. (2006), many local extinction events due to climate change may have been missed because of lack of attention to small-scale detail. Hybridization may be overlooked for the same reasons. Given that the majority of studies identifying new hybrid zone formation have been published within the last 5 years (Table 1), it seems likely that many more examples of recent hybrid zone formation have yet to be identified.

### Temporal isolation

The specific timing of life-history events such as reproduction (i.e., phenology) is often influenced by climate. The effect of climate change on phenology is already being observed in numerous taxa (Cleland et al. 2007; Parmesan 2007). If one species advances its reproductive period (e.g., flowering time) into the window of a second sympatric species that either does not respond, or that responds in a different direction, to changing climate, hybridization may occur. A shift in the timing of breeding events has been documented in several species from a wide range of taxa including frogs (Carroll et al. 2009), birds, (Goodenough et al. 2010), and plants (Menzel et al. 2006). The actual effect of climate change on phenology is, however, highly species-specific (Parmesan 2007). For example, Crimmins et al. (2010) have found some plant species have shifted to earlier flowering times with increasing temperatures, while other species are shifting flowering times in the opposite direction with concurrent decreases in precipitation. This work highlights the point that the response to climate change is complex, species-specific, and may be dependent on *both* shifts in temperature and precipitation regimes.

Consequently, the degree of reproductive synchrony between sympatric congeners may be altered by shifting climate. Evidence suggests that climate change will strongly affect reproductive overlap between sympatric species. For example, recent experimental work in 12 tallgrass prairie species has found that artificial warming results in increased periods of reproductive overlap between some competing species pairs (Sherry et al. 2007). Similarly, field observations have identified that delayed breeding by some species and advanced breeding by other amphibian species has increased the period of temporal overlap since 1979 at one wetland in South Carolina (Todd et al. 2010).

Shifting phenologies may also increase hybridization rates in current hybrid zones. For example, temporal differences in flowering maintain species barriers between two ash species (*Fraxinus excelsior x F. angustifolia*) (Gerard et al. 2006). Occasional hybrids do, however, occur, and the flowering time of hybrids is intermediate to that of parental species. Gerard et al. (2006) predict that increasing temperatures will extend the reproductive overlap between these species, resulting in one species expanding its range at the expense of the other.

### Behavioral isolation

**Figure 1:**
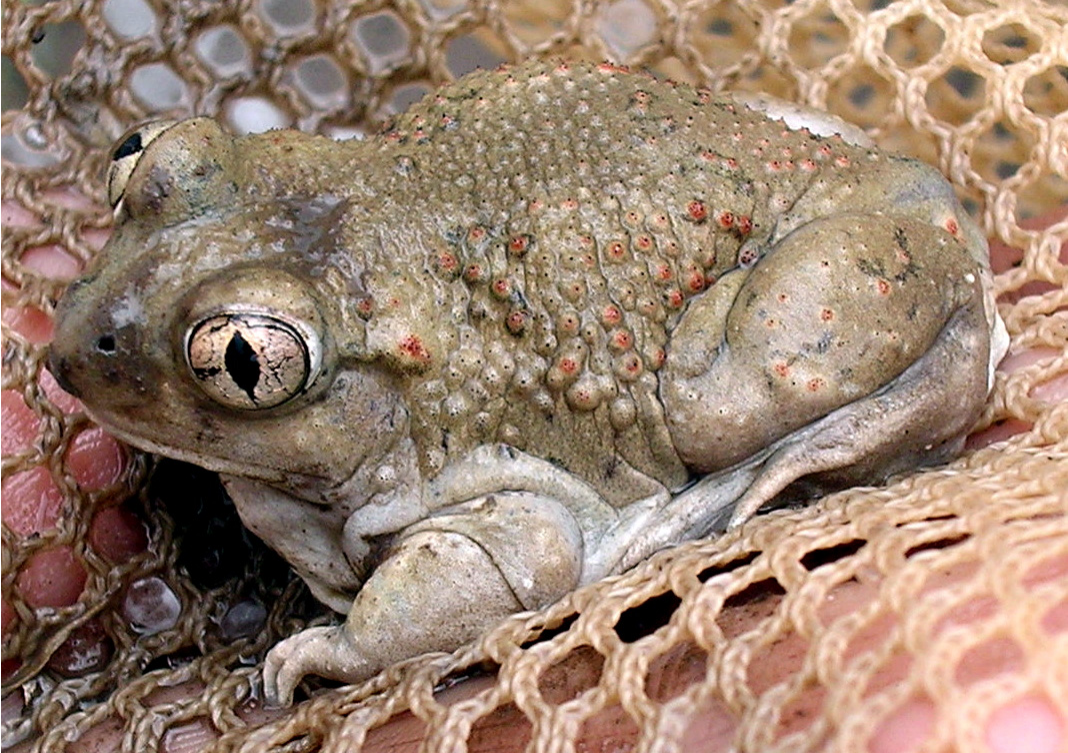
A Mexican spadefoot toad (*Spea multiplicata*). Hybridization between Mexican and Plains spadefoot toads is predicted to increase due to the changing climate in the American southwest. Photo credit: Dr. Amber Rice.

Fundamentally, hybridization is a phenomenon resulting from mate choice. In some species, females use environmental cues during mate choice, causing the frequency of hybridization to be highly dependent on the abiotic environment (e.g., Pfennig 2007). Shifting climates will drastically alter these environmental signals, potentially resulting in environments where hybridization is more likely. For example, the frequency of hybrids between Mexican spadefoot toads (*Spea multiplicata*) (Fig. 1) and Plains spadefoot toads (*Spea bombifrons*) is inversely correlated with the size of the ephemeral ponds in which they breed (Pfennig and Simovich 2002). Experimental work has shown that this pattern can be explained by the increased preference for heterospecific Mexican spadefoot toad (*Spea multiplicata*) males expressed by female Plains spadefoot toads in low water conditions (Pfennig 2007). Because hybrid offspring develop faster relative to pure species, hybridization is adaptive in ponds that would dry before pure species offspring completed metamorphosis (Pfennig and Simovich 2002). As pond depth is affected by temperature and precipitation, the hotter, drier summers predicted for the western United States where the spadefoot toad hybrid zone is located (Seager et al. 2007) may result in increased hybridization between these species (Chunco et al. 2012).

Reproductive behavior is also strongly influenced by the availability of suitable mates. Both males and females of many species are more likely to engage in heterospecific matings if conspecific mates are rare (Hubbs 1955; Avise and Saunders 1984; Wirtz 1999). Climate change may directly impact mate availability by two mechanisms. First, relative frequencies of species may change due to climate-mediated range shifts or differential effects of climate on population dynamics. Climate change may alter the species composition within a community if one species becomes either increasingly rare (e.g., one species is disproportionately negatively affected by shifting climates) or increasingly common (e.g., via a novel colonization event as new habitat becomes available). Climate change is currently affecting population dynamics, as some species are experiencing local population declines (Thomas et al. 2006), while in other species, new populations are being established as ranges expand (Parmesan 2006). These changing species ratios are already leading to new cases of hybridization. For example, hybridization between brown hares (*Lepus europaeus*) and mountain hares (*L. timidus*) has been documented at the northern extent of the range of brown hares, and hybridization frequency is correlated with the relative abundance of each species (Jansson et al. 2007). Specifically, hybridization occurs more frequently in areas most recently colonized by brown hares, as brown hare densities are still low at this expanding range edge (Jansson et al. 2007). Because this range expansion is correlated with a recent series of milder winters (Jansson and Pehrson 2007), climate change appears to be driving the formation of this new hybrid zone. In this case, spatial barriers were overcome by a climate-mediated range expansion, while low mate availability at the expansion front resulted in heterospecific matings. Thus, although here each reproductive barrier has been presented independently, multiple barriers will often be simultaneously overcome by climate change and thus multiple mechanisms will work in concert to promote hybridization.

Climate change is also predicted to increase the range overlap between willow grouse (*Lagopus lagopus*) and rock ptarmigan (*L. muta*) in Scandinavia (Quintela et al. 2010). Consequently, the frequency of hybridization between females of the rarer species (rock ptarmigan) and males of the more common species (willow grouse) will likely increase (Quintela et al. 2010). Interestingly in this latter case, the more common species (willow grouse) is also larger in body size than the rarer species (Quintela et al. 2010). As females in many different taxa prefer larger males (Andersson 1994), some species may be especially predisposed to hybridize when secondary contact occurs between two populations that differ in average body size.

Second, climate change may also alter mate availability via effects on sex ratio. If sex ratios deviate from 50:50, hybridization frequency can increase because suitable conspecific mates are rare (Lushai et al. 2005; Cordingley et al. 2009). This can result either from females being more likely to accept heterospecific mates when conspecific males are rare (Wellenreuther et al. 2010) or by an increase in forced copulations by males when conspecific females are rare (Evans and Magurran 1999). This is a particular issue for species with temperature-dependent sex determination (Hulin et al. 2009). In these species, sex ratios are correlated with local climatic conditions (Wapstra et al. 2009). Janzen (1994) suggest that a temperature increase of as little as 4 degrees C is enough to eliminate the production of male offspring in painted turtles (*Chrysemys picta*). The temperatures at some nesting beaches used by several species of marine turtles (a group in which all species use temperature-dependent sex determination) are currently high enough to yield heavily female-biased clutches (Hays et al. 2003; Glen and Mrosovsky 2004). In marine turtles, viable hybrids have been reported between four of the five genera in the family Cheloniidae, despite the fact that these lineages are highly divergent (i.e., species in hybrid pairs were estimated to have diverged 10–75 Mya) (Karl et al. 1995). Even in some species with heteromorphic sex chromosomes, temperature may over-ride genetically determined sex, resulting in skewed sex ratios (e.g., Shine et al. 2002). Bickford et al. (2010) estimate that all Southeast Asian reptiles and most amphibians are at risk for sex ratio skews severe enough to threaten population viability within 100 years.

## PostMating Barriers

In addition to altering opportunities for hybridization to occur, climate change can also alter the outcome of hybridization by influencing the relative fitness of pure species and hybrids. Hybrid fitness is frequently environmentally dependent, with hybrids outcompeting parental species in some environments, but not others (Arnold 1997). The specific dynamics of hybrid fitness will thus be highly sensitive to environmental change.

For example, hybrids between *Daphnia galeata* and *D. hyalina* perform well under stressed conditions relative to pure species (Keller et al. 2008). Additional work has shown an increase in the abundance of *D. galeata x D. hyalina* following an ice-free winter compared with past colder winters (Zeis et al. 2010). Thus, future warming and the resultant increase in frequency in ice-free periods will likely greatly alter the relative abundances of pure species and hybrids. In hybrid offspring between an invasive species (the tiger salamander, *Ambystoma tigrinum*) and a native species (the California tiger salamander, *A. californiense*), temperature is shown to have an impact on locomotion, suggesting that future climate change may facilitate the spread of the hybrid swarm (Johnson et al. 2010).

To date, the majority of work on climate-mediated hybridization has focused on the increased opportunities for hybridization to occur. Yet, as climate change will inevitably alter selection, the relative fitness of pure species and hybrids may be altered as well.

## Evolutionary Consequences

Hybridization has had a much greater role in the evolutionary lineage of plant and animal species than previously recognized. Current estimates suggest that approximately 25% of plant and 10% of animal species naturally hybridize (Mallet 2005). Ultimately, the consequences of hybridization on the evolution of a species will depend both on the relative fitness of hybrid offspring compared with pure species offspring and on the frequency of hybrid matings. As with pure species, hybrid fitness depends on genotype by environment interactions (Arnold and Hodges 1995). These interactions are, however, complex and could result in several alternative outcomes including: (1) extinction of one of the parental species due to wasted mating effort (Rhymer and Simberloff 1996) or genetic swamping (Seehausen et al. 2008; Brumfield 2010); (2) the reinforcement of species boundaries (Blair 1955; Servedio and Noor 2003); (3) the creation of a third, hybrid, species (Rieseberg 1997); (4) the formation of a stable hybrid zone (Arnold 1997); or (5) partial introgression between the two hybridizing lineages (Taylor et al. 2009; Fitzpatrick et al. 2010).

Predicting which of the above outcomes will be most common when species hybridize as a result of climate-mediated secondary contact is difficult given the current lack of data. However, parallels to the invasive species literature provide some insight into the relative likelihood of each outcome. Although novel interactions resulting from climate change are not a result of species invasion in any traditional sense, the situations are similar in that, in both cases, two formerly isolated taxa are brought into secondary contact as a direct or indirect result of human action. At the same time, climate change may facilitate the invasion process (Walther et al. 2009). The study of hybridization between invasive and native species is significantly more mature than the study of climate-mediated hybridization (Rhymer and Simberloff 1996; Vellend et al. 2007). It can thus offer valuable lessons.

First, hybridization can exacerbate extinction risk for many species. Currently, hybridization between native and invasive species is a major conservation concern (Allendorf et al. 2001), and examples of taxa at risk of extinction as a result of hybridization with an invasive species abound in the literature (Rhymer and Simberloff 1996). Hybridization can cause extinction with remarkable rapidity; theoretical work suggests that extinction can occur in as little as five generations (Wolf et al. 2001). As backcrossing tends to occur from rare species to more common species (Lepais et al. 2009), extinction seems particularly likely when one species is already relatively rare. Second, introgression is likely to be a lasting legacy of climate-mediated hybridization. Theoretical work shows that even infrequent hybrid matings can lead to extensive introgression (Barton 2001). At the extreme end, introgression can lead to a loss of novel genetic lineages (Mooney and Cleland 2001). However, this outcome is not inevitable. Indeed, when invasive and native species hybridize, partial introgression, where both parental taxa retain distinct features, occurs quite commonly (e.g., Fitzpatrick et al. 2010). Third, hybrid offspring may outcompete pure species, particularly in novel environments, thus promoting further invasion. For example, Hovick et al. (2012) show that hybrids between wild (*Raphanus raphanistrum*) and cultivated (*Raphanus sativus*) radishes can perform better in a novel environment well outside the current invasive range.

Climate change, invasion biology, and hybridization are inexorably linked. Because the climate is changing so rapidly, current habitat will soon represent novel environments for the species living there. If hybrids outperform parental species in these novel environments, further hybridization and accelerated range expansions by southern species into the territories of northern species may result, exacerbating the invasion process.

Climate-mediated hybridization will differ from that involving invasive species in one important way, however. While invasions result in novel species interactions, climate change will result in novel species interactions that also occur within the context of novel environments. Climate change is creating environments unlike any seen on Earth in recent history (Saxon et al. 2005; MacDonald 2010). This change in habitat is a major threat to current biodiversity (Thomas et al. 2004). Theoretical results suggest that hybridization can rescue populations otherwise at risk of extinction following environmental change (Baskett and Gomulkiewicz 2011). Empirically, many hybrid taxa show high fitness in novel or extreme environments where parental fitness is poor. For example, a butterfly species of hybrid origin is associated with an extreme alpine environment (Gompert et al. 2006). Also, three hybrid sunflower species (*Helianthus anomalus*, *H. deserticola*, and *H. paradoxus*) are found in more extreme environments than any parental species (Rieseberg et al. 2007). Finally, hybridization may facilitate the presence of one species of spadefoot toad (*Spea bombifrons*) in environments that would otherwise be too arid for population persistence (Chunco et al. 2012).

This pattern of high fitness in extreme environments will be particularly important in responding to climate change. For example, hybrid corals frequently colonize marginal habitats at the periphery of parental ranges, suggesting that hybridization might be a major factor in driving range expansion and is likely to be particularly important as a means of adapting to climate change (Willis et al. 2006). Seehausen (2004) has also argued that hybridization can facilitate adaptive radiations in habitats that are disturbed or novel. Climate-mediated hybridization therefore has the potential to trigger an adaptive radiation in certain lineages.

Hybridization is an important creative force in evolution (Anderson and Stebbins 1954; Arnold 1997) and a key means of exposing a wide range of genetic and phenotypic variation to selection (Arnold and Martin 2010). Unlike mutation, hybridization simultaneously results in novel variation in multiple genes within a single generation (Rieseberg et al. 2003; Seehausen 2004). Furthermore, hybridization commonly results in offspring with phenotypic traits that are more extreme than seen in either parental lineage (i.e., transgressive segregation, Rieseberg et al. 1999, 2003). Indeed, a meta-analysis found transgressive traits reported in 91% of the included studies (Rieseberg et al. 1999). The increase in phenotypic variation resulting from hybridization can promote adaptation in both the parental habitats (Barton 2001) and in novel environments (Lewontin and Birch 1966; Rieseberg et al. 2007).

Hybridization also plays a key role in speciation (Abbott et al. 2013). Whether the rate of evolutionary adaptation is sufficient to keep pace with the rate of predicted environmental change is currently an open question for many taxa. Because many populations have low potential to adapt to climate change considering only within-species responses (Hoffmann and Sgro 2011), hybridization may be an important mechanism for allowing population persistence for some species in the face of rapid climate change.

## Future Directions

Climate change will undoubtedly alter the likelihood of hybridization. While increasing habitat fragmentation (Tilman et al. 1994) and range retractions (Thomas et al. 2006) may isolate some populations and thus reduce the likelihood of hybridization in those taxa, I argue here that, because of the reasons mentioned above, the net balance of hybridization in the future will tip toward increasing hybridization frequency. Currently, several novel climate-mediated hybrid zones have been reported (Table 1). Very little work to date has, however, explicitly looked at changes in hybrid zone dynamics under climate change. As climate change progresses, there will be many avenues of study in several different areas that merit attention. Below, I outline four lines of inquiry that will be particularly informative.

First, ecological niche models are already widely used to predict how species' distributions will change under different climate change scenarios (e.g., Elith et al. 2010; Thuiller et al. 2011). Extending this work to predict where new contact zones will form will identify regions and taxa where climate-mediated hybridization is most likely to occur as a result of the dissolution of habitat barriers. Currently, most examples are novel hybrid zone formation are from northern latitudes or mountainous regions in the northern hemisphere. Less work has focused on climate-mediated hybrid zone dynamics in tropical or subtropical regions or the southern hemisphere. Using multispecies biogeographic models to look for kinds of regional patterns in predicted novel hybrid zones would be particularly useful in guiding future empirical work. Along the same lines, comparing the relative role of climate compared with other drivers of hybridization at a global scale will help refine predictions of the role climate change will play in altering hybrid zone dynamics.

Second, the National Phenology Network is accumulating large amounts of data on reproductive timing for a wide range of taxa (http://www.usanpn.org/). This database could be used to identify sympatric species pairs that are becoming more similar in reproductive timing and hence overcoming temporal barriers. As above, this work would begin to identify both taxa and regions where climate-mediated hybridization is most likely to occur. Both the biogeographic models above and phenological data can also be used to establish more definitively whether hybridization will be more likely in the future.

Third, translocation and common garden experiments will be a way of empirically testing whether species will breed and the resulting fitness of their hybrid offspring if brought into secondary contact. For example, Wellenreuther et al. (2010) found that, in one species of damselflies (*Calopteryx virgo*), males from northern allopatric populations have partially lost the ability to discriminate between conspecific and heterospecific females, suggesting that if these populations come into secondary contact as a result of climate change, hybridization will result.

Finally, genetic studies have already revealed a recent case of climate-mediated hybridization (Garroway et al. 2010). Using markers to identify hybrids in new contact zones will be an important step in establishing the scope and degree of hybridization during initial secondary contact. Further, using phylogeographic tools to link past periods of climate change to current genetic diversity can shed light on how historic periods of rapid climate change have previously influenced hybrid zone dynamics and thus allow better predictions of the outcomes of current shifts in community composition.

Understanding how climate change may affect hybridization is critically important in predicting community responses to climate change. Yet, this remains challenging as many studies have reported idiosyncratic responses to climate change among even closely related species (Moritz et al. 2008; Tingley et al. 2009). Incorporating interspecific reproductive interactions may partially explain nonintuitive differences in species' responses to climate change. For example, because the geographic locations of tension hybrid zones are strongly dependent on gene flow and relatively independent of environmental conditions (Barton 1979; Barton and Hewitt 1985), hybridization could prevent further range expansion and even result in range retractions or other nonintuitive range shifts. As an example, a hybrid zone between two warbler species (*Dendroica townsendi x D. occidentalis*) has recently (i.e., within the last ∼15 years) shifted southwards with the southward range expansion of *D. towndsendi*, counter to climate change prediction (Krosby and Rohwer 2010).

Additionally, climate-mediated hybridization might allow us to observe the evolutionary dynamics of hybridization in situ. Currently, empirical studies of hybridization frequently rely on well-established hybrid zones (Rand and Harrison 1989; Yanchukov et al. 2006) or experimental crosses between hybridizing lineages (Rieseberg et al. 2003; Donovan et al. 2009). Climate change will provide an opportunity to identify new sites where secondary contact is occurring and to examine the consequences of natural hybridization over time.

Recent work has estimated that a vast proportion of the Earth's biodiversity may be susceptible to hybridization, particularly as a result of human disturbance (Seehausen et al. 2008). Given this, it is essential to consider the possibility of climate-mediated novel reproductive interactions before hybridization occurs unchecked, and unobserved, in the wild.
